# Displacement of intraorbital ferromagnetic foreign bodies induced by magnetic resonance imaging: Quantification using an animal model

**DOI:** 10.1016/j.redii.2025.100064

**Published:** 2026-01-15

**Authors:** Camille Cathelineau, Marwane Ghemame, Antoine Le Boëdec, Béatrice Carsin-Nicol, Hervé Saint-Jalmes, Pierre-Antoine Éliat, Frédéric Mouriaux, Jean-Christophe Ferré

**Affiliations:** a Service de radiologie et imagerie médicale, centre hospitalier universitaire de Rennes, université de Rennes, 35000 Rennes, France; b Service d'ophtalmologie, centre hospitalier universitaire de Rennes, université de Rennes, 35000 Rennes, France; c Université de Rennes, centre hospitalier universitaire de Rennes, Inserm, LTSI - UMR_S 1099, 35000 Rennes, France; d Université de Rennes, CNRS, Inserm, Biosit- UMS 3480, US_S 018, Prism, 35000 Rennes, France; e Université de Rennes, Inrae, Inserm, institut Numecan - UMR_A 1341, UMR_S 1241, 35000 Rennes, France

**Keywords:** Safety, MRI, Foreign body, Steel ball, Eye, Orbit

## Abstract

**Rationale and objectives:**

The presence of ferromagnetic foreign bodies in the intraorbital area, eyelids, or intraorbital fat of an examined subject represents a contraindication to magnetic resonance imaging (MRI). We sought to measure the displacement of one or two intraocular and intraorbital ferromagnetic foreign bodies in ex vivo porcine heads following 1.5 T MRI scan compared to 5-min walk test.

**Materials and methods:**

In this ex-vivo controlled laboratory study, a total of 48 1-, 2-, and 4-mm steel balls were surgically implanted into suprachoroidal, intraorbital fat, intrapalpebral, and intravitreal locations of 36 fresh porcine heads. Ferromagnetic foreign bodies displacement was measured by comparing computed tomography scan before and after both a 5-min walk test to simulate handling-related movement and 1.5 T MRI scan. Comparison of groups was performed using the non-parametric Mann-Whitney-Wilcoxon test.

**Results:**

Global median displacement after 5-min walk test was 0.8 mm. Compared to the control group, the ferromagnetic foreign bodies displacement after MRI was significant for the suprachoroidal location with single ferromagnetic foreign body (2.3 mm, *p* = 0.0282), and for the intravitreal location with single ferromagnetic foreign body (4.5 mm, *p* = 0.0282) and double ferromagnetic foreign bodies (5.0 mm, *p* = 0.0282). Compared to the control group, ferromagnetic foreign body displacement after MRI was significant for the double 2-mm ferromagnetic foreign bodies (2.2 mm, *p* = 0.0130), single 4-mm ferromagnetic foreign body (4.9 mm, *p* = 0.0107), and double 4-mm ferromagnetic foreign bodies (9.0 mm, *p* = 0.0084).

**Conclusion:**

Ferromagnetic foreign body displacements after 1.5 T magnetic field exposition were not significant for extraocular intrapalpebral and intraorbital fat locations. Neither were there significant displacements for 1-mm ferromagnetic foreign body, irrespective of the location. On the other hand, the presence of two objects increased the displacement of 2- and 4-mm ferromagnetic foreign bodies.

## 1 Introduction

Magnetic resonance imaging (MRI) is widely used for diagnostic, research, interventional, and surgical purposes. During MRI diagnostic examinations, patients and healthcare professionals in the immediate equipment environment can be exposed to three variants of magnetic fields, including static magnetic fields (B0), time-varying magnetic field gradients, and radiofrequency magnetic fields (B1). Currently, commercially available clinical systems range from 0.2 to 3 T. Certain 7 T scanners are mainly used for research purposes, in addition to emergent clinical applications. Most scanners installed for general diagnostic purposes are 1.5 T in strength. This strong magnetic field may cause undesirable effects, such as biological effects, acoustic noise, or attractive forces [1,2]. The presence of ferromagnetic foreign bodies in the intraorbital area, eyelids, or intraorbital fat still represents a contraindication to MRI. Indeed, ferromagnetic foreign bodies can cause several injuries, due to the projectile effect, torque effect, and potential surrounding heat [3].

Only very few studies have so far examined intraorbital ferromagnetic foreign body movements due to MRI, reporting contradictory results depending on their size or location [[4], [5], [6], [7], [8]]. Moreover, the presence of several ferromagnetic foreign bodies could theoretically modify their movements on account of their attractive or repulsive effects due to the induced magnetization of the object located within a magnetic field. To the best of our knowledge, no study to date has examined this effect.

Based on a previously described ex vivo animal model [9], this study sought to measure using computed tomography (CT) the 1.5 T MRI-induced displacement of intraorbital ferromagnetic foreign bodies, depending on their number (one or two), size, and location, compared to 5-min walk test.

## 2 Methods

### 2.1 Animal model

We used a previously described ex vivo animal model that was able to detect steel ball movements induced by MRI [9].

A fresh porcine model was chosen because of its anatomical similarity to humans. We used quarters of porcine heads, each of which comprised one whole orbit with a globe, orbital fat, muscles, and eyelids. Ferromagnetic foreign bodies were implanted within 2 days, and images were acquired within 5 days postslaughter. Porcine heads were maintained in a refrigerator at 4°C, without freezing between manipulations, to ensure preservation of soft-tissue properties [10], [11]. Each porcine head was wedged in an airtight container after surgical implantation of one or two calibrated ferromagnetic foreign bodies so as to facilitate manipulation and orientation during imaging.

All applicable international, national, and institutional guidelines for the care and use of animals before slaughter were followed. This study used bovine tissue obtained from animals slaughtered for the human food chain. No animals were sacrificed specifically for this research. Therefore, in accordance with EU Directive 2010/63/EU and French regulations on animal experimentation, no ethical approval was required.

### 2.2 Ferromagnetic foreign bodies

A total of 48 steel balls were implanted in 36 porcine heads, as detailed in Table 1.

**Table 1:** Size, number, and location of ferromagnetic foreign bodies (steel balls) implanted in porcine heads.

Size	1-mm		2-mm		4-mm	
Number of implanted ferromagnetic foreign bodies	1	2	1	2	1	2
Suprachoroidal	2	2	2	2	2	2
Intraorbital fat	2	2	2	2	2	2
Intrapalpebral	2	2	2	2	2	2
Intravitreal	2	2	2	2	2	2

We used calibrated steel balls with at least 98 % of iron (Duratool steel balls, Farnell, UK) that were surgically implanted into four locations, as previously described [9].

### 2.3 Imaging acquisitions

The imaging protocol included two or three CT acquisitions (CT scan 1 to 3) and one MRI session. CT and MRI scanners were located in the same hospital, separated by a 5-min walk.

All CT scans were performed using a 64-detector row Aquilion PRIME scanner (Canon medical systems, Otawara, Japan) with the following parameters: 120 kV with automatic mAs modulation, 0.75 s rotation time, 0.5 mm collimation, and 0.625 mm spiral pitch factor. Image reconstruction was performed with a hard reconstruction kernel (FC35), resulting in 244 slices with a 0.8 mm gap and an image matrix of 512 × 512 with voxel sizes of 0.3 × 0.3 × 1 mm. Porcine heads were always positioned the same way in the airtight container: snout ahead, eye looking up. Airtight containers containing the implanted porcine heads wedged with pads were carefully positioned using the CT scanner laser.

MRI scans were performed on a 1.5 T scanner (Optima MR450w General Electric Medical System Milwaukee, Wisconsin), using a 24-channel head coil. Conventional MRI sequences including three-dimensional (3D) T1 fast spin echo (FSE), 3D T1 enhanced fast gradient echo (EFGRE), 3D T2 FSE, 3D susceptibility-weighted angiography (SWAN) EFGRE and 2D EPI b1000 diffusion-weighted imaging acquisitions were performed to mimic a typical patient examination, with a total acquisition time of 20 min. Specific absorption rates were respectively 1.202, 0.163, 0.274, 0.055, 0.049 W/kg. The airtight container was positioned at the center of the head coil, wedged with pads. Its orientation was the same as for CT scans. Indeed, the container was carefully introduced into and removed from the magnetic field core in the same way and at the same speed.

All porcine heads underwent at least CT scan 1, MRI scan, and CT scan 3. To assess the potential displacements due to head’s handling between examinations, half of the heads additionally underwent a 5-min walk test with stairs and CT scan 2 before MRI scan.

The total scheme comprising three CT scans and one MRI scan lasted less than 1.5 h.

### 2.4 Image analyses

All CT and MRI images were transferred to the local PACS TELEMIS® TM system (Version 4.80, Louvain-la-Neuve, Belgium) and to a personal computer.

Ferromagnetic foreign body location was verified on CT scan 1 images visualized on the PACS and compared to the theoretical surgical implantation. MRI scans were analyzed to confirm the ferromagnetic nature of the foreign bodies with the presence of magnetic susceptibility artifacts. Ferromagnetic foreign body displacement due to head’s handling between examinations (5-min walk test) was measured by comparing CT scan 2 to CT scan 1, while their displacement due to the MRI scan was measured by comparing CT scan 3 to CT scan 2 or 1 (without 5-min walk test).

ITK-SNAP® (Version 3.8.0, Pennsylvania, USA), an open source software, was used to compare CT scans and measure FFB displacement.

For ferromagnetic foreign body displacement measurement, an automatic rigid registration was initially carried out between the two CT 3D image volumes. A radiologist visually checked the registration based on undeformable structures. If automatic registration was not satisfactory, we completed it with manual registration using sinus walls, orbital cortical bones, and ocular muscles as anatomical landmarks. The locations of the ferromagnetic foreing body center were then determined on both volumes to measure mean ball displacement in three directions using the Pythagoras formula: *d* = $\sqrt{a^{2}+b^{2}+c^{2}}$ (*d*: final, *a*: axial, *b*: coronal, *c*: sagittal displacements).

### 2.5 Statistics

The control group was composed of the porcine heads that underwent the 5-min walk test, representing half of the total number of porcine heads.

The control group was compared to the experimental group according to ball size or location, with an additional distinction made between single- and double ferromagnetic foreign bodies for the analyses.

Quantitative results were expressed as median displacement [1^st^ percentile; 3^rd^ percentile]. Comparison of groups (experimental versus control) was carried out using the non-parametric Mann-Whitney Wilcoxon test. For all statistical tests, the significance threshold was set at *p* < 0.05. Statistical analyzes were performed using the SAS software, v.9.4® (SAS Institute, Cary, NC, USA).

## 3 Results

**Fig. 1:**
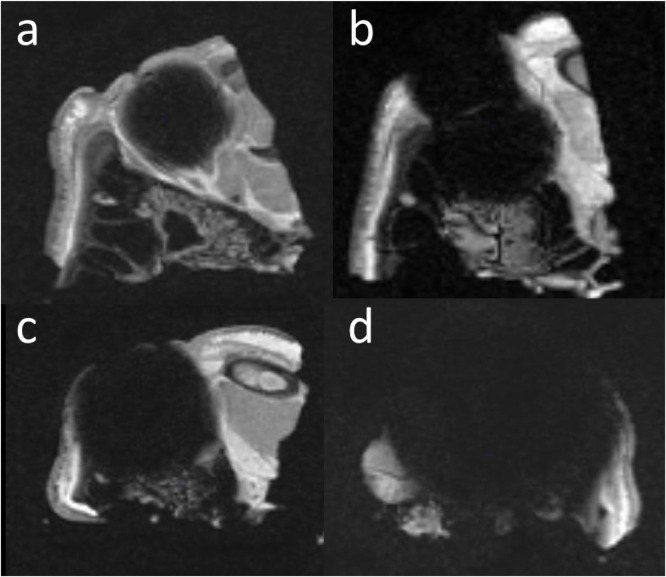
Examples of magnetic susceptibility artifacts on MRI. a: 1-mm ferromagnetic foreign body; b: two 1-mm ferromagnetic foreign bodies; c: 2 mm ferromagnetic foreign body; d: 4 mm ferromagnetic foreign body.

CT scan 1 confirmed the implanted location of the 48 ferromagnetic foreign body in 36 porcine heads. All MRI scans demonstrated pronounced susceptibility artifact consistent with ferromagnetic material (Fig. 1).

### 3.1 Control group

Global median displacement following the 5-min walk test was 0.7 mm [0.6; 0.8], with a median displacement of 0.8 mm [0.7; 1.1] for single- and of 0.6 mm [0.4; 0.8] for double ferromagnetic foreign bodies.

Median ferromagnetic foreign bodies displacements in the control group according to their location are reported in Table 2.

**Table 2:** Displacement of single and double ferromagnetic foreign bodies according to their location.

	Single ferromagnetic foreign body				Double ferromagnetic foreign bodies		
Location		Control group	Experimental group	*p*	Control group	Experimental group	*p*
Suprachoroidal		*n* = 3 1.0 [0.8; 0.8; 1.1; 1.1]	*n* = 6 2.3* [1.3; 1.6; 6.8; 16.8]	*p* = 0.0282	*n* = 3 0.7 [0.3; 0.3; 0.8; 0.8]	*n* = 6 3.3 [0.5; 0.9; 11.4; 22.1]	*p* = 0.0933
Intraorbital fat		*n* = 3 0.7 [0.5; 0.5; 0.7; 0.7]	*n* = 6 2.8 [0.2; 0.5; 5.7; 5.9]	*p* = 0.4347	*n* = 3 0.6 [0.3; 0.3; 0.7;0.7]	*n* = 6 1.6 [0.4; 0.5; 4.8; 6.6]	*p* = 0.2453
Intrapalpebral		*n* = 3 1.3 [0.8; 0.8; 1.5. 1.5]	*n* = 6 1.5 [0.4; 0.6; 1.7; 5.6]	*p* = 0.7954	*n* = 3 0.6 [0.5; 0.5; 1.0; 1.0]	*n* = 6 1.9 [0.3; 0.8; 2.2; 2.3]	*p* = 0.2453
Intravitreal		*n* = 3 0.6 [0.4; 0.4; 0.8; 0.8]	*n* = 6 4.6* [1.5; 2.2; 5.8; 9.9]	*p* = 0.0282	*n* = 3 0.6 [0.3; 0.3; 0.8; 0.8]	*n* = 6 5.0* [1.9; 4.5; 19; 19.2]	*p* = 0.0282

Quantitative results are expressed as median displacement in mm [min; Q1; Q3; max]. Significant *p* values < 0.05 are shown with *.

Median displacements of ferromagnetic foreign bodies in the control group according to their size are reported in Table 3.

**Table 3:** Displacement of single and double ferromagnetic foreign bodies according to their size.

	Single ferromagnetic foreign body				Double ferromagnetic foreing bodies		
Ball size		Control group	Experimental group	*p*	Control group	Experimental group	*p*
1-mm		*n* = 4 0.9 [0.6 ; 0.7; 1.2 ; 1.3]	*n* = 8 2.0 [0.2; 1.0; 6.3; 9.9]	*p* = 0.2027	*n* = 4 0.3 [0.3 ; 0.3; 0.7 ; 1]	*n* = 8 0.8 [0.3; 0.5; 1.8. 4.5]	*p* = 0.0833
2-mm		*n* = 4 0.8 [0.5; 0.7; 1.0; 1.1]	*n* = 8 1.4 [0.4; 0.9; 2.3; 5.7]	*p* = 0.1727	*n* = 4 0.8 [0.6; 0.7; 0.8; 0.8]	*n* = 8 2.2* [0.8; 0.9; 5.0; 5.5]	*p* = 0.013
4-mm		*n* = 4 0.8 [0.4; 0.6; 1.2; 1.5]	*n* = 8 4.9* [1.5; 2.7; 5.8; 16.8]	*p* = 0.0107	*n* = 4 0.6 [0.5; 0.6; 0.7 ; 0.7]	*n* = 8 9.0* [2.2; 3.6; 19.1; 22.1]	*p* = 0.0084

Quantitative results are expressed as median displacement in mm [min; Q1; Q3; max]. Significant *p* values < 0.05 are shown with *.

### 3.2 Experimental group

Despite some displacements, all of the 48 ferromagnetic foreign bodies remained located in their original anatomical region on CT scan 3.

Global median displacement following the MRI scan was 2.2 mm [1.1; 5.6], with a median displacement of 2.2 mm [1.4; 5.7] for single- and of 2.2 mm [0.9; 5.3] for double ferromagnetic foreign bodies.

Median displacements after MRI for single and double ferromagnetic foreign bodies according to their location and size are presented in Table 2, Table 3.

**Fig. 2:**
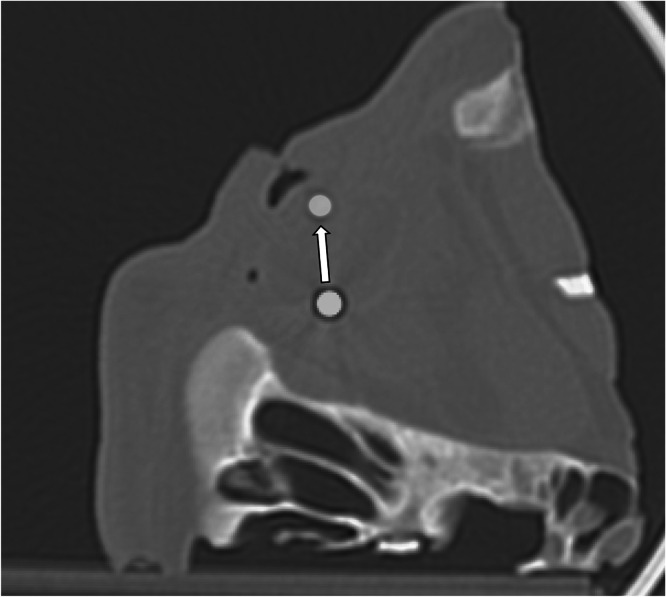
Fusion of pre- and post-MRI CT scans after rigid spatial registration–coronal view. The white arrow shows the displacement of an intravitreal 4-mm ferromagnetic foreign body after MRI scan.

Compared to the control group, ferromagnetic foreign body displacement following MRI scan was significant for the suprachoroidal location with single onject (2.3 mm [1.6; 6.8]), as well as for the intravitreal location with single and double objects (4.5 mm [2.2; 5.8] and 5.0 mm [4.5; 19]), respectively. For both single and double ferromagnetic foreign bodies, intraorbital fat and intrapalpebral locations did not demonstrate any significant displacement. Fig. 2 illustrates the displacement of a 4-mm ferromagnetic foreign body in the intravitreal location.

**Fig. 3:**
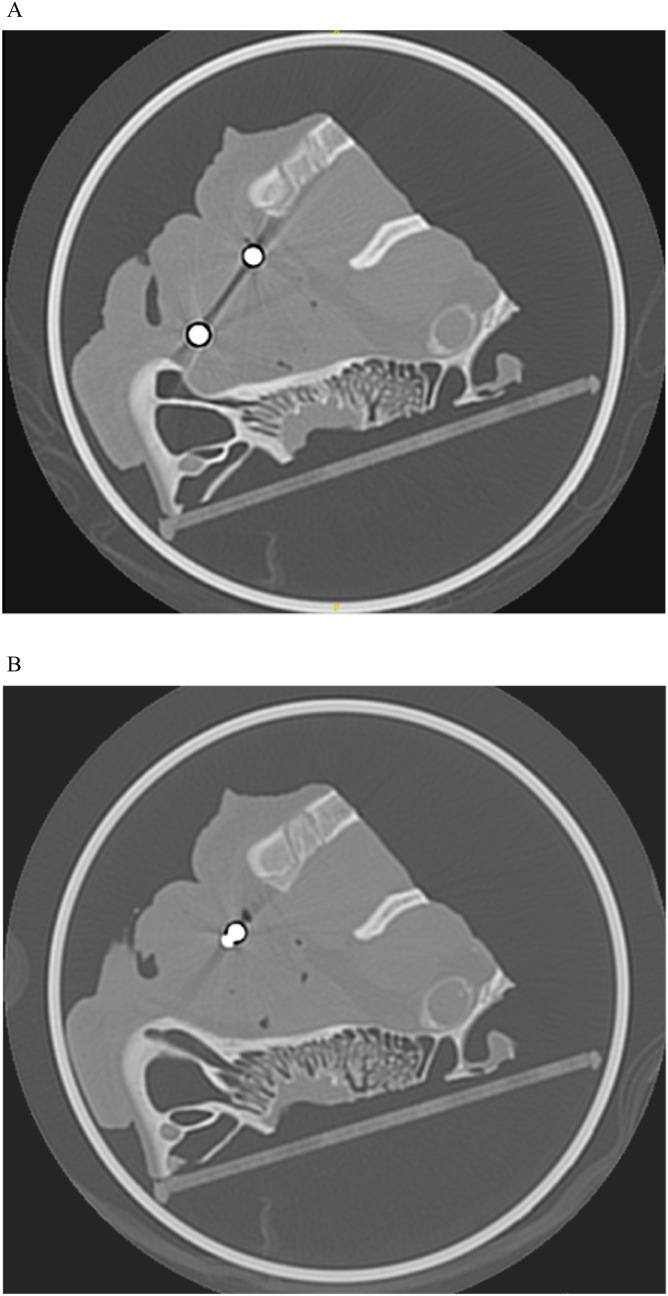
The pre- (a) and post-MRI (b) CT scans of two intravitreal 4-mm ferromagnetic foreign bodies show the post-MRI displacement including an attractive effect.

Compared to the control group, ferromagnetic foreign body displacement following MRI scan was significant for the double 2-mm (2.2 mm [0.9; 5.0]), for the single 4-mm (4.9 mm [2.7; 5.8]), and for the double 4-mm objects (9.0 mm [3.6; 19.1]). No significant displacement was observed for 1-mm ferromagnetic foreign body. The attractive forces between double 4-mm ferromagnetic foreign bodies are illustrated in Fig. 3.

## 4 Discussion

In this study, we have shown that displacements of intraorbital ferromagnetic foreign bodies following MRI examination depended on their location, number, and size. The median displacement following 5-min walk test was 0.8 mm. Compared to the 5-min walk test, displacements after 1.5 T MRI examination were significant (i) for the suprachoroidal location with single ferromagnetic foreign body, (ii) for the intravitreal location with single and double objects, as well as (iii) for the double 2-mm, single 4-mm, and double 4-mm ferromagnetic foreign bodies.

The prevalence of metallic foreign bodies in the orbits of patients undergoing MRI is low. Williamson et al. found a prevalence of 0.27 % using plain radiography and/or CT scans in at-risk patients [12]. Some clinical damages were reported, such as local pain, hyphema, or increased ocular pressure [[13], [14], [15], [16], [17], [18]]. This risk could be accounted for by several factors, including foreign body location, size, number, form, and composition. However, the risk of clinical damages is probably low when a metallic foreign body is present in the orbits [12,19,20].

Our study showed a significant displacement of intravitreal and suprachoroidal ferromagnetic foreign bodies, i.e. intraocular objects. By contrast, intrapalpebral and intraorbital fat ferromagnetic foreign bodies did not show any significant displacement, although the displacements were greater than those observed after the 5-min walk test. Whatever the displacement, objects remained in their initial anatomical region after MRI scan. The MRI-induced displacement of intraocular ferromagnetic foreign body was described in vivo and in vitro in the 1980s. Lagouros et al. reported substantial retinal injuries with three of four in vivo intravitreal ferromagnetic foreign bodies [5,8]. As for Williams et al., using an in vivo rabbit model, they demonstrated ferromagnetic foreign body displacement after 2.0 T MRI only with the largest intravitreal object (3 × 1 × 1 mm) [6]. Gunenc et al. implanted four ferromagnetic foreign bodies into intravitreal or suprachoroidal locations of fresh bovine eyes [8]. They demonstrated movement of all objects after 1.0 T MRI, ranging from 7 mm to 10 mm, and mentioned ocular damages yet without providing a precise description.

The 1- and 2-mm ferromagnetic foreign body displacements following MRI were not significant as compared to the 5-min test. They were lower than that observed with 4-mm ferromagnetic foreign bodies. Irrespective of the number of ferromagnetic foreign bodies, displacements of 1-mm objects were not significant. Several studies have shown that ferromagnetic foreign body movements mainly appeared with high size objects, notably with 3-mm objects in an in vivo rabbit model at 2.0 T and with 2-mm objects [6], [8]. Moreover, Zhang et al. reported two patients with infra 0.5-mm ferromagnetic foreign bodies, discovered during MRI examination and confirmed by ophthalmic surgery, with no evidence of MRI-induced intraocular damages [19]. In fact, even though 2 mm may seem insignificant, such a movement would have an impact if the ferromagnetic foreign body may be recent and close to the sclera because intraorbital fat movement in MRI probably occurs less often because of tissue fibrosis that encapsulates foreign bodies in granulomas [9]

We showed that the displacement of double 2-mm and 4-mm ferromagnetic foreign bodies was a least 1.7-fold that of single 2-mm and 4-mm objects. This effect was not visible for 1-mm ferromagnetic foreign bodies. As shown in Fig. 3, this could be explained by the induced magnetization attraction between ferromagnetic foreign bodies, which may not have been enough to induce increased displacement for small objects. Indeed, in a sufficiently strong external magnetic field, ferromagnetic bodies acquire an induced magnetization aligned with the field. The resulting dipolar magnetic fields create a non-uniform local field, giving rise to mutual forces described by the dipole–dipole interaction, which typically leads to attraction. Because multiple ferromagnetic foreign bodies are frequent, 22.4 % according to Zhang et al., this ex vivo observation could be of relevance for better understanding MRI-induced ocular lesions [21].

We chose steel balls with at least 98 % of iron to assess the maximal effect of ferromagnetic displacements. Magnetic intraocular foreign bodies are frequent: 53.48 % according to Zhang et al. [21]. Previous studies found no displacement with non-ferromagnetic foreign bodies, including diamagnetic components, such as glass or wood, or paramagnetic metal, such as copper or aluminum [5,8]. The use of ball-type objects did not permit to study the torque effect, which tends to align a ferromagnetic foreign body with the magnetic field. It is best visualized with oblong objects, in particular needles or stylets. Cullen et al. showed a change of angulation following 1.5 T MRI of an intraocular 3-mm stylet ferromagnetic foreign body implanted in vivo in rabbits [4]. According to magnetic field intensity and ferromagnetic foreign body location, we can suppose that this effect could injure some parts of the eye.

### 4.1 Limitations

Our study has some limitations. We studied ferromagnetic foreign body displacement before and after MRI examination. While the effect of magnetic field is stronger inside the magnet, for example for the torque effect, the magnetic field gradient during the in- or outgoing magnetic field could expose to complications. However, it is technically impossible to explore the effects during installation and it is very difficult to analyze ferromagnetic foreign body movement during MRI due to the important susceptibility artifacts. Although the number of implanted ferromagnetic foreign bodies was moderate, some statistically significant results were obtained, in line with the literature.

The ex vivo model has the advantage of facilitating the manipulations. However, this model is not suitable to study medium- and long-term changes, in particular the fibrotic processes around the ferromagnetic foreign body, such as described by Winter et al. [22]. Cullen et al. reported that the angular motion of an in vivo rabbit intraocular 3-mm stylet ferromagnetic foreign body was lower on day 30 than on the implantation day, whereas distance displacement did not significantly differ [4]. The ex vivo model is probably more sensitive to detect displacements. Potential undesirable effects of ferromagnetic foreign body in a high radiofrequency magnetic field include those related to heating. We did not explore this limited effect. Dedini et al. demonstrated that the temperature of bullets (longer than 12 mm) increased up to 0.2°C above background heating at 3 T [23]. We used spherical ferromagnetic foreign bodies that do not allow the measurement of torque effects. Moreover, the protocol used does not permit exploration of the respective roles of static and fringe-field gradients encountered during entry into the MRI bore.

We implanted ferromagnetic foreign bodies in four locations, including two intraocular locations, but not in the anterior segment, which represents around 21 % of intraocular foreign bodies [21].

We chose to study the effect of 1.5 T magnetic field because it is the most widely used. However, 3T MRI is currently being increasingly used, in particular for neuroimaging. Further studies are thus needed to assess the latter magnetic field strength.

## 5 Conclusion

In conclusion, this ex vivo model helped to demonstrate the displacements of ferromagnetic foreign bodies after 1.5 T magnetic field exposition, compared to the 5-min walk test. These displacements were variable according to ferromagnetic foreign body size and location. Significant displacements of intraorbital and intraocular- as well as 2- and 4-mm ferromagnetic foreign bodies were shown. However, these displacements were not significant for extraocular, intrapalpebral, and intraorbital ferromagnetic foreign bodies, and for 1-mm objects irrespective of the location. Our work support that the risk is low in these cases, therefore we could recommend not contra-indicating 1.5 T MRI examinations in these patients.

## CRediT authorship contribution statement

**Camille Cathelineau:** Conceptualization, Methodology, Investigation, Formal analysis, Writing – original draft. **Marwane Ghemame:** Conceptualization, Methodology, Investigation, Formal analysis, Writing – original draft. **Antoine Le Boëdec:** Formal analysis, Writing – original draft. **Béatrice Carsin-Nicol:** Conceptualization, Methodology. **Hervé Saint-Jalmes:** Conceptualization, Methodology. **Pierre-Antoine Éliat:** Conceptualization, Methodology, Investigation, Writing – original draft, Writing – review & editing, Supervision. **Frédéric Mouriaux:** Conceptualization, Methodology, Writing – review & editing, Supervision. **Jean-Christophe Ferré:** Conceptualization, Methodology, Writing – review & editing, Supervision.

## Declaration of competing interest

The authors declare that they have no known competing financial interests or personal relationships that could have appeared to influence the work reported in this paper.
